# Consumption of antibiotics in Chinese public general tertiary hospitals (2011-2014): Trends, pattern changes and regional differences

**DOI:** 10.1371/journal.pone.0196668

**Published:** 2018-05-03

**Authors:** Xiaoyuan Qu, Chang Yin, Xihong Sun, Shusheng Huang, Chaofan Li, Panpan Dong, Xiufang Lu, Zhuo Zhang, Aitian Yin

**Affiliations:** 1 School of Health Care Management, Key Laboratory of Health Economics and Policy Research, NHFPS, Shandong University, Jinan, Shandong, China; 2 Information Center, National Institute of Hospital Administration, Beijing, China; Azienda Ospedaliera Universitaria di Perugia, ITALY

## Abstract

**Background:**

China has a high rate of antibiotic use. The Chinese Ministry of Health (MOH) established the Center for Antibacterial Surveillance (CAS) to monitor the use of antibacterial agents in hospitals in 2005. The purpose of this study was to identify trends, pattern changes and regional differences in antibiotic consumption in 151 public general tertiary hospitals across China from 2011–2014.

**Materials and methods:**

Valid data for antibiotic use were collected quarterly, and the antibiotic consumption data were expressed as the defined daily dose (DDD) per 100 inpatient days (ID). We compared the patterns of antibiotic use in different classes and geographical clusters.

**Results:**

Total antibiotic use significantly decreased (P = 0.018) from 75.86 DDD/100 ID in 2011 to 47.03 DDD/100 ID in 2014. The total consumption of flomoxef sodium and cefminox increased from 1.31 DDD/100 BD in 2011 to 8.6 DDD/100 BD in 2014. Cephalosporins were the most frequently used antibiotics in all regions. Third-generation cephalosporins accounted for more than 45% of the cephalosporins used. Carbapenem use substantially increased (P = 0.043). Penicillin combinations with inhibitors accounted for 50% of the penicillin used, and prescribed meropenem accounted for most of the carbapenems used in all regions in 2014. The subclasses in each antibiotic group were used differently between the seven regions, and the total hospital antibiotic use in 2014 differed significantly by region (P = 0.014).

**Conclusion:**

Although the volume and intensity of total antibiotic use decreased, the antibiotic use patterns were not optimal, and broad-spectrum antibiotics were still the main classes. The aggregate data obtained during the study period reveal similar antibiotic consumption patterns in different regions. These findings provide useful information for improving the rational use of antibiotics. More detailed data on antibiotics linked to inpatient diseases need to be collected in future studies.

## Introduction

Antibiotic consumption is one of the main causes of bacterial resistance to antibiotics and is a major global public health problem[1], particularly in China[2]. China is estimated to be the second largest consumer of antibiotics worldwide. An increase in antibiotic consumption of up to 57% between 2000 and 2010 in the hospital sectors in Brazil, Russia, India, China and South Africa (i.e., the BRICS countries) was reported to be attributable to China[3]. An epidemiological study reported that extended-spectrum β-lactamase (ESBL)-producing *Enterobacteriaceae*, methicillin-resistant *Staphylococcus aureus* (MRSA) and carbapenem-resistant *Acinetobacter baumannii*, represent more than 50% of microbial isolates in China[4].

Several factors contribute to the misuse of antibiotics in China. First, physicians might overprescribe antibiotics because of a lack of knowledge about rational use or because they want to prevent potential infections. Second, antibiotics are regarded as a panacea or cure-all by patients, so they demand them even when they are not indicated, and they may also ask for newer antibiotics because they think these will be more effective[5]. Third, expenditures on drugs as a proportion of total healthcare costs are high. Most public hospitals earn 90% of their revenue from the services they provide. Providers are allowed to charge a 15% mark-up on drugs; thus, physicians are incentivized to prescribe drugs that are not clinically needed because their bonuses are frequently tied to these revenues. Drug revenues accounted for 41.4% of the total hospital revenues in 2011[6].

To change this situation, promote the rational use of antimicrobial agents, contain antimicrobial resistance, support the global governance endeavours of the World Health Organization (WHO) [7]and comply with new healthcare reforms introduced since 2009, the Chinese National Health and Family Planning Commission (NHFPC, i.e., the former Ministry of Health) implemented a 3-year national campaign to control the inappropriate use of antibiotics in various types of medical institutions at all levels on 1 July 2011, with a primary focus on public secondary and tertiary hospitals[8]. This campaign mainly consisted of establishing mandatory management strategies for the rational use of antimicrobials, setting targets for antimicrobial management, organizing a task force, developing audit and inspection systems and investigating and reassigning responsibilities for hospital management staff who violate rational use policies. According to the policies, all hospitals should form an antibiotic administrative group and enforce formulary restrictions (i.e., antibiotic procurement should be restricted to 50 agents in tertiary hospitals). Moreover, targets of antibiotic prescription for hospitalized patients are set at less than 60%, prophylactic use of antibiotics in clean operations should be lowered to 30% of patients and total inpatient consumption of antibiotics should be less than 40 defined daily doses/100 inpatient days[9].

In 2005, the Chinese Ministry of Health (MOH) established the Center for Antibacterial Surveillance (CAS) to monitor the use of antibacterial agents in hospitals in China (expect Hong Kong SAR, Macau SAR and Taiwan Province)[10]. The CAS is a national network of surveillance systems that collects data quarterly, aims to collect comparable and reliable data on antibiotic use in hospital care in China, and provides technical support for the promotion of the rational use of antimicrobial agents. The CAS has a sound organizational management structure, a convenient data submission/reporting system and a strict quality management system[11].

Some countries use national surveillance data to monitor antibiotic use. In Europe, retrospective data collected by the European Surveillance of Antibiotic Consumption (ESAC) project revealed a rather constant antibiotic consumption pattern with high variation across countries[12]. Data collected by the Japanese Antimicrobial Consumption Surveillance (JACS) project found that consumption of antimicrobials increased from 2009 to 2013[13]. Data on antibiotic use in the Republic of Korea collected by the National Health Insurance system showed significantly increased consumption of third-generation cephalosporins, carbapenems, and glycopeptides from 2008 to 2012[14]. Studies in China have collected data from hospitals’ information systems and medical procurement administrative agencies, finding that outpatient and inpatient antibiotic use, antibiotic prescription rates, inpatient parenteral antibiotic use, patient-given antibiotic prophylaxis for > 24 h and patient costs for hospital stays decreased significantly after 2011 interventions[7, 15–17].

These studies in China focused on a small number of hospitals or cities, did not analyse overall or subgroup comparisons of antibiotic use between different years and did not assess the effects of the interventions on changes in the use of different drugs in different regions. Studies investigating the differences in antibiotic use must be conducted in different regions of China, which has a population approaching 1.4 billion and a vast area. Therefore, in this study, we attempted to complete the following goals: first, we aimed to investigate the trends in antibiotic consumption by inpatients in public tertiary hospitals in China after the 3-year national campaign; second, we aimed to explore the variations in the consumption and patterns of antibiotic use in different regions; and third, we aimed to analyse the evolution of frequently used antibiotics and hospital-specific antibiotics (mainly the carbapenems and glycopeptides) in 151 hospitals between 2011 and 2014. In contrast to previous studies, our study is based on the national surveillance database, which contains reliable and valid data with more extensive coverage. Hence, this study is complementary and supplementary to previous studies and provides baseline information to improve future antimicrobial stewardship programmes.

## Materials and methods

### Data collection

The CAS currently has 192 core member institutions, which are public tertiary hospitals (including 181 general hospitals and 11 specialized hospitals) in 31 provinces, autonomous regions and municipalities in mainland China[11]. In this study, we analysed antibiotic consumption data from 151 public general hospitals in 30 provinces, autonomous regions and municipalities in mainland China (except for hospitals in Tibet) from the core member institutions. The 151 participating hospitals serve as regional medical centres, and each hospital has at least 2000 beds.

This study was a retrospective analysis. The data regarding antibiotic consumption by inpatients were collected quarterly from 2011–2014 from the CAS and did not include discharge medication. The data contained information for the following variables: region (provinces, autonomous regions and municipalities); hospital name; quarter; drug specification; unit; generic name; quantity of consumption; and inpatient days. The data on inpatient days were calculated by multiplying the quarterly total number of hospital discharges with the mean number of days of hospitalization[16].

The validated data were managed in Microsoft Excel 2016 (Microsoft Corporation, Redmond, USA). Aggregated surveillance data for antibiotic consumption were limited to systemic antibacterial agents and classified according to the Anatomical Therapeutic Chemical (ATC) classification (WHO, version 2015). The data were collected at the level of the active ingredient (level 5 of ATC class J01) and the defined daily dose (DDD) measurement unit. The number of DDDs (defined daily doses) was calculated according to the WHO Collaborating Centre for Drug Statistics Methodology (available at http://www.whocc.no/atc_ddd_index/). The antibiotic consumption data were divided into six main antibiotic groups, including the penicillins and beta-lactamase inhibitors (J01C), cephalosporins+carbapenems+monobactams (J01D), macrolides and lincosamides (J01F), quinolones (J01M), glycopeptides+polymyxins+steroid antibacterial+imidazole derivatives+nitrofuran derivatives (J01X) and other antibiotics (including tetracyclines, amphenicols, sulphonamides, trimethoprim and aminoglycoside antibacterial agents and J01A+J01B+J01E+J01G). The glycopeptides (J01XA) and carbapenems (J01DH) are regarded as the last resort against serious bacterial resistance[3] are and mainly used in tertiary hospitals in China; thus, we explored the characteristics of their use separately.

The penicillins (J01C) were further divided into the following four subclasses: narrow-spectrum penicillins (J01CE); broad-spectrum penicillins (J01CA); beta-lactamase-resistant penicillins (J01CF); and combinations with beta-lactamase inhibitors (J01CR). The cephalosporins were further divided into the following five subclasses: the four generations and other cephalosporins (J01DI, primarily cefminox). The glycopeptides (J01XA) were further divided into the following two subclasses: vancomycin (J01XA01) and teicoplanin (J01XA02). The carbapenems (J01DH) were further divided into the following five subclasses: imipenem and cilastatin (J01DH51); panipenem and betamipron (J01DH55); meropenem (J01DH02); faropenem (J01DH03); and biapenem (J01DH05).

The participating hospitals in the different provinces, autonomous regions and municipalities were geographically clustered into the following seven regions: north China (NC); east China (EC); south China (SC); central China (CC); northeast China (NEC); northwest China (NWC); and southwest China (SWC). The distribution of the 151 hospitals in the different regions is shown in Table 1.

**Table 1:** Distribution of hospitals in the different regions across China.

NEC N = 12	NC N = 32	EC N = 31	CC N = 22	SC N = 27	SWC N = 11	NWC N = 16
Liaoning Province (4)	Beijing Municipality(18)	Shanghai Municipality (8)	Henan Province (10)	Guangdong Province (19)	Chongqing Municipality (2)	Shaanxi Province (4)
Jinlin Province (4)	Tianjin Municipality (2)	Jiangsu Province (4)	Hubei Province (8)	Guangxi autonomous region (4)	Sichuan Province (5)	Gansu Province (3)
Heilongjiang Province (4)	Hebei Province (7)	Zhejiang Province (4)	Hunan Province (4)	Hainan Province (4)	Guizhou Province (1)	Qinghai Province (2)
	Shanxi Province (3)	Anhui Province (3)			Yunnan Province (3)	Ningxia autonomousregion (3)
	Inner Mongolia (2)	Fujian Province (3)				Xinjiang autonomousregion (4)
		Shandong Province (6)				
		Jiangxi Province (3)				

### Data analysis

The inpatient antibiotic consumption was measured using DDDs per 100 inpatient days (DDD/100 ID). The overall and subclass comparisons of antibiotic consumption between 2011 and 2014 were performed using the non-parametric Wilcoxon signed-rank test. The significance threshold was set at 5% (P<0.05). Antibiotic consumption in hospitals in the different regions was compared using the non-parametric Kruskal-Wallis test with a two-sided P-value <0.05 as the significance level. The percentages of the subclasses for each of the main antibiotic classes were compared among the seven regions. The data were analysed using the statistical package IBM^®^ SPSS^®^ Statistics 20.

## Results

### Antibiotic use trends

#### Total use

**Fig 1:**
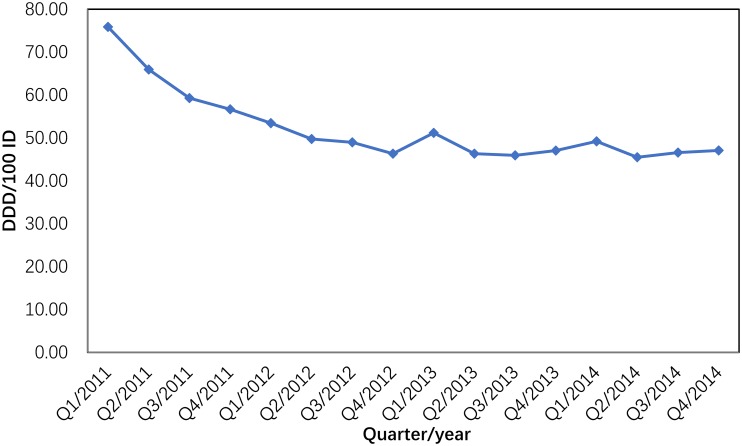
Total antibiotic consumption in 151 hospitals in China (2011–2014).

Between 2011 and 2014, antibiotic consumption in the participating hospitals expressed as the total number of DDDs decreased by 9.31% (from 58,177,380.9 to 52,763,728.92 DDDs) as the total number of inpatient days increased during this period by 18.98% (from 90,898,598.66 to 112,191,721.2). The total antibiotic consumption trends are shown in Fig 1. The largest decrease was observed between quarter 1 of 2011 and quarter 4 of 2012: it fell from 75.86 DDD/100 ID to 46.32 DDD/100 ID (38.94% reduction) and then plateaued. Between quarter 1 of 2011 and quarter 4 of 2014, the antibiotic consumption decreased by 37.93% (Fig 1).

#### Evolution of regional antibiotic consumption

Table 2 the proportion of the major antibiotic groups used remained relatively stable in all regions. Over the 4-year period, the consumption of antibiotics showed a significant reduction (P = 0.018) in all areas. The cephalosporins (J01D) were the most frequently used antibiotics in all areas in 2014, followed by the penicillins (J01C), quinolones (J01M) and other antibacterial agents (J01X). The total variation observed in the hospital antibiotic consumption in all areas in China ranged from 41.52 DDD/100 ID in NWC to 50.22 DDD/100 ID in SC.

**Table 2:** Hospital antibiotic use in China (evolution between 2011 and 2014).

	DDD/100 ID		J01C %		J01D %		J01F %		J01M %		J01X %		J01A/B/E/G %	
	2011	2014	2011	2014	2011	2014	2011	2014	2011	2014	2011	2014	2011	2014
**Overall**	64.00	47.03	11.42	13.23	55.97	52.36	4.3	6.68	13.39	12.46	7.05	8.83	7.16	6.43
**NC**	65.71	44.50	12.78	12.51	49.68	52.06	10.23	6.11	13.09	10.65	8.15	7.52	6.06	12.56
**EC**	70.64	47.08	13.59	14.15	50.90	51.75	5.44	3.88	12.86	16.74	10.66	8.88	6.56	4.60
**CC**	66.97	48.35	13.72	14.96	54.51	50.74	5.65	6.19	11.60	14.39	8.51	8.74	6.02	4.97
**SC**	62.65	50.22	10.15	12.69	54.95	55.83	6.98	5.01	12.31	12.74	7.97	7.86	7.64	5.87
**NEC**	61.67	49.59	10.57	13.50	53.24	55.10	8.32	6.64	11.67	10.59	9.65	8.91	6.38	5.27
**SWC**	59.28	42.24	15.64	17.14	51.99	54.08	4.84	4.16	11.92	13.10	9.29	7.16	6.33	4.36
**NWC**	57.20	41.52	12.20	13.33	56.01	58.6	6.95	4.33	10.91	11.73	8.39	6.84	5.54	5.17

### Evolution of the use of the main classes of antibiotics

#### Use of cephalosporins

**Fig 2:**
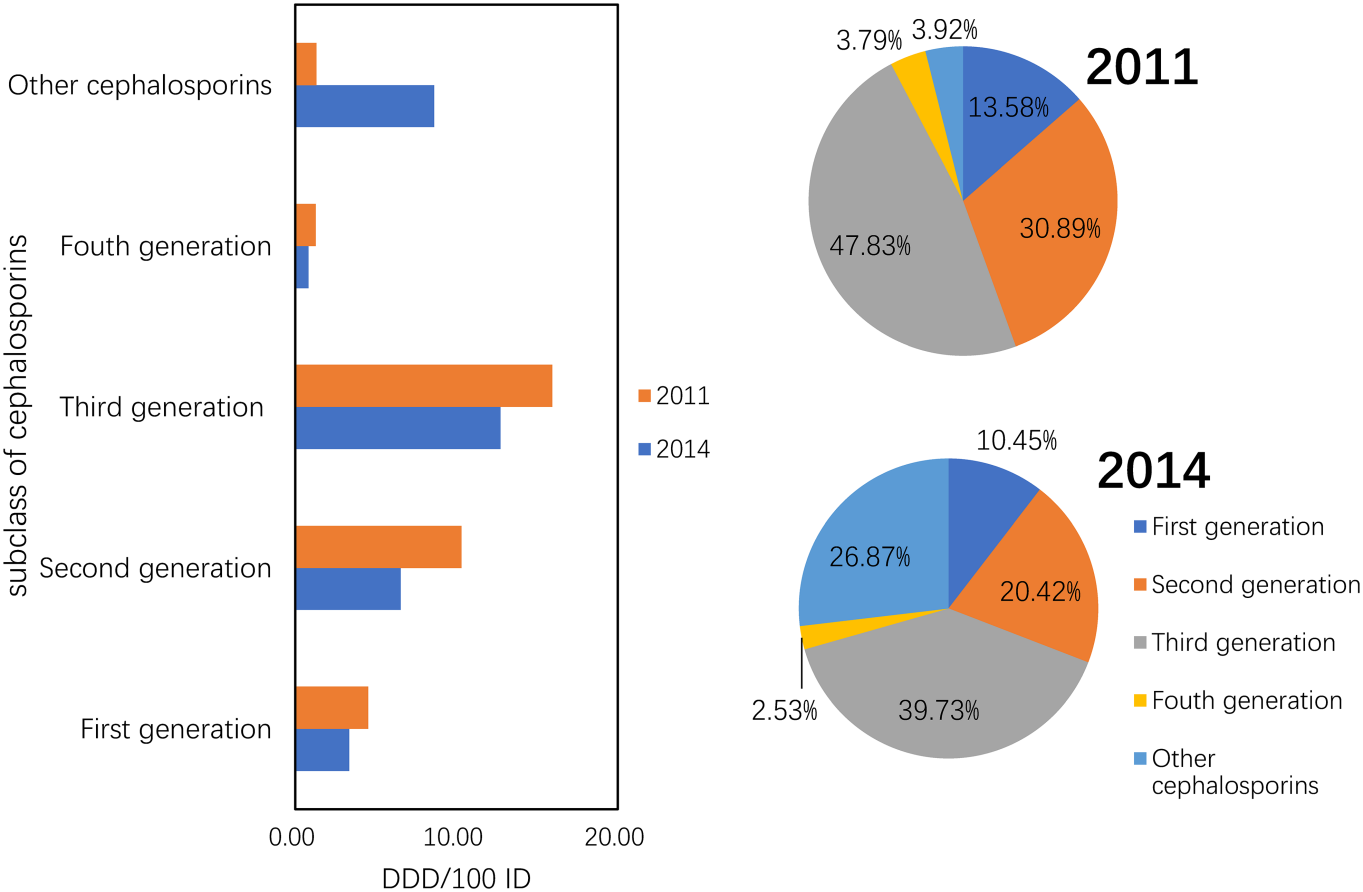
Changes in the patterns of cephalosporin use between 2011 and 2014 (n = 151 hospitals).

The consumption of the other cephalosporins increased by 6.56-fold from 1.31 DDD/100 ID in 2011 to 8.6 DDD/100 ID in 2014. Their proportions increased from 3.92% to 26.87%, whereas the consumption of the first-, second-, third- and fourth-generation cephalosporins was reduced by 26.13%, 36.53%, 20.23% and 35.96%, respectively (P = 0.05) (Fig 2).

#### Use of penicillins (J01C)

**Fig 3:**
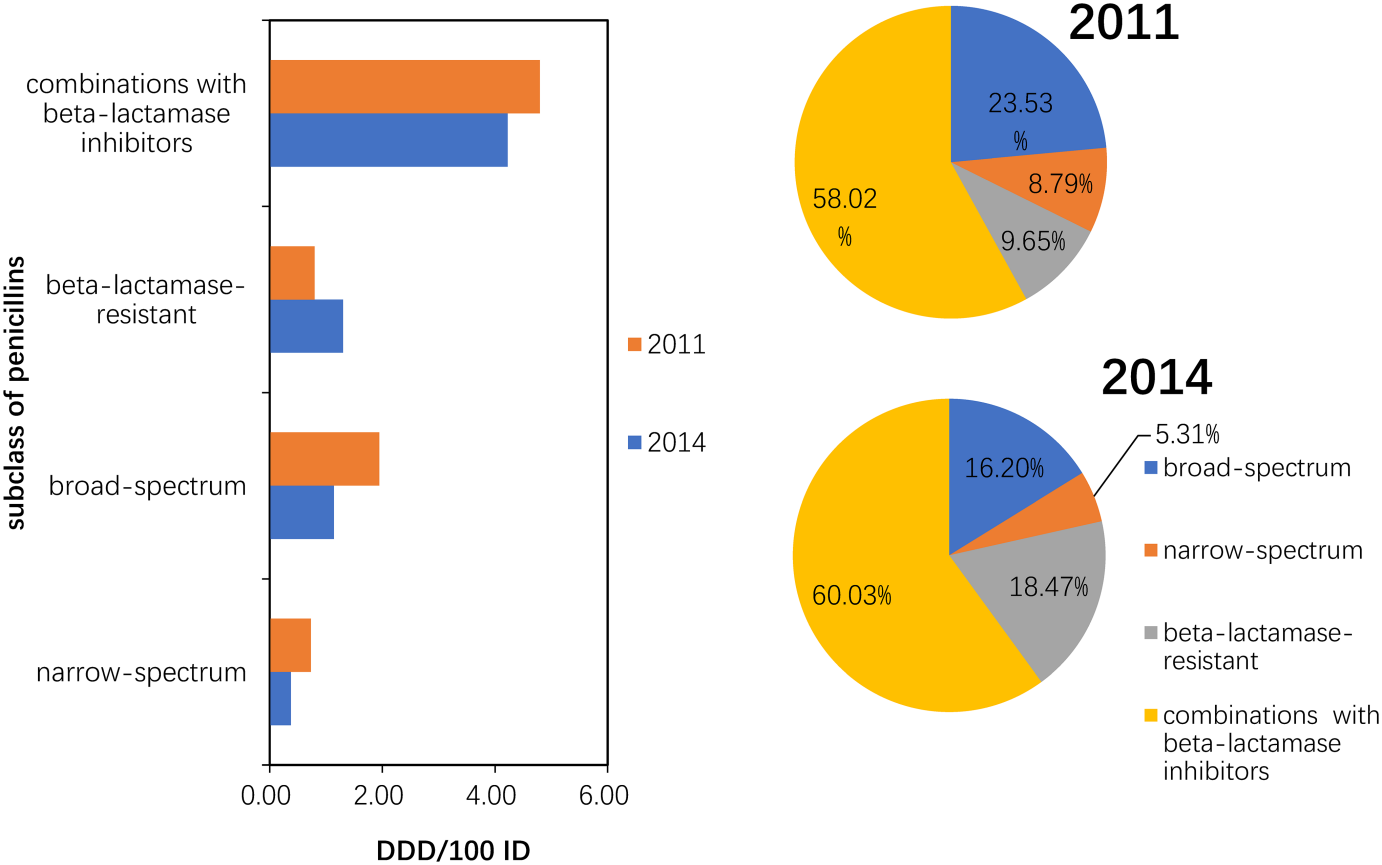
Changes in the patterns of penicillin use in hospital care between 2011 and 2014 (n = 151 hospitals).

Between 2011 and 2014, the consumption of narrow-spectrum, broad-spectrum and combinations with beta-lactamase inhibitor penicillins was reduced by 49.32%, 41.23% and 11.90%, respectively. Compared to 2011, the 2014 consumption pattern showed a proportional increase in beta-lactamase-resistant penicillin and combinations with beta-lactamase inhibitors that was compensated by the decrease in broad-spectrum and narrow-spectrum penicillins (P = 0.10) (Fig 3).

#### Use of quinolones (J01MA+MB)

**Fig 4:**
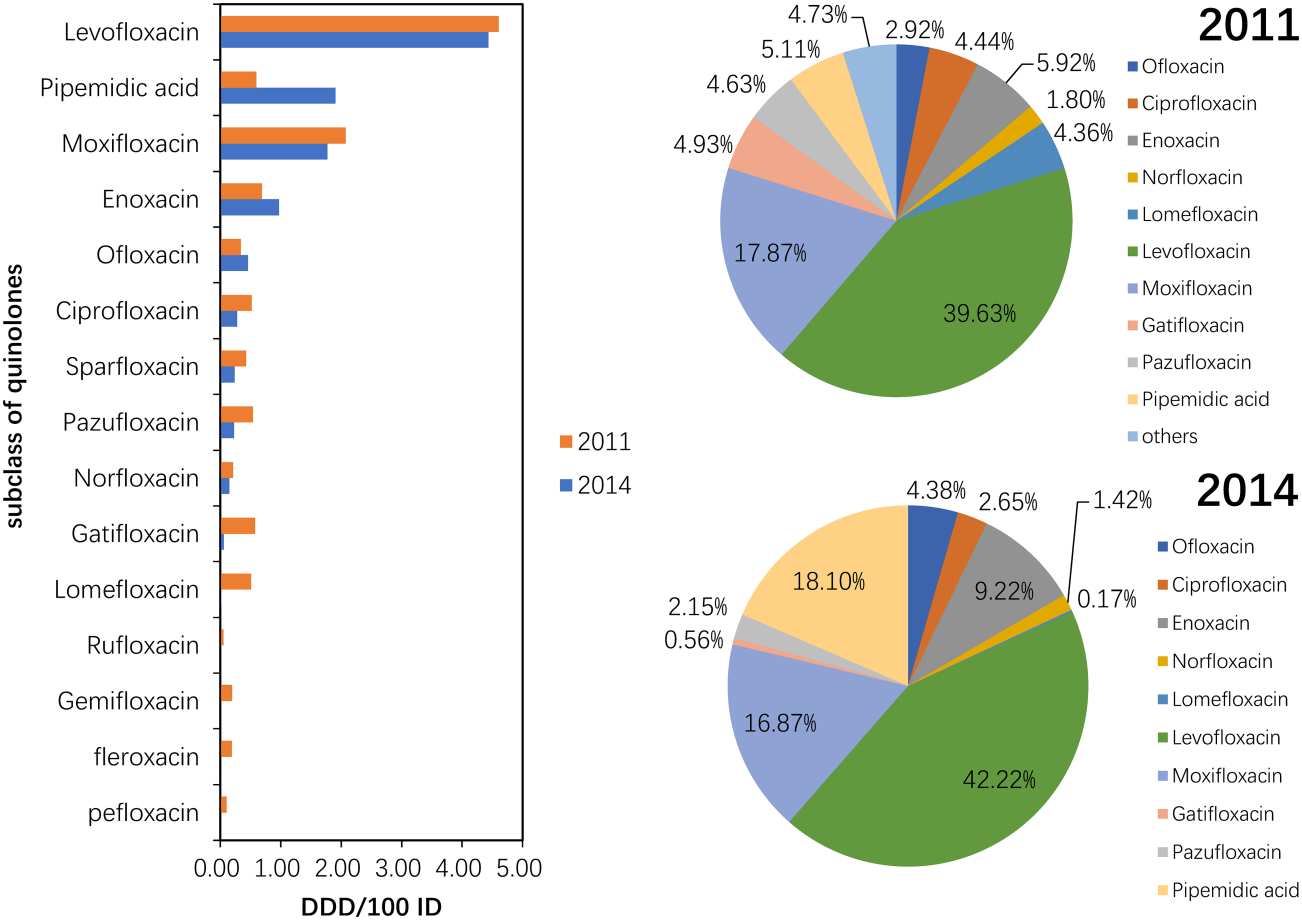
Changes in the patterns of quinolone use in hospital care between 2011 and 2014 (n = 151 hospitals).

Although these drugs were used in 2011, pefloxacin, fleroxacin, rufloxacin and gemifloxacin were not used in the participating hospitals in 2014. The consumption of pipemidic acid showed a large (more than 100%) increase (222.03%) (P = 0.317). The proportion of pipemidic acid use accounted for 18.10% of the total quinolone consumption in 2014, whereas this drug accounted for only 5.11% of the total quinolone consumption in 2011 (P = 0.078). The most frequently used class of quinolones in 2011 and 2014 was levofloxacin, which accounted for more than 30% of the total quinolone consumption each year (Fig 4).

#### Use of carbapenems (J01DH) and glycopeptides (J01XA)

**Fig 5:**
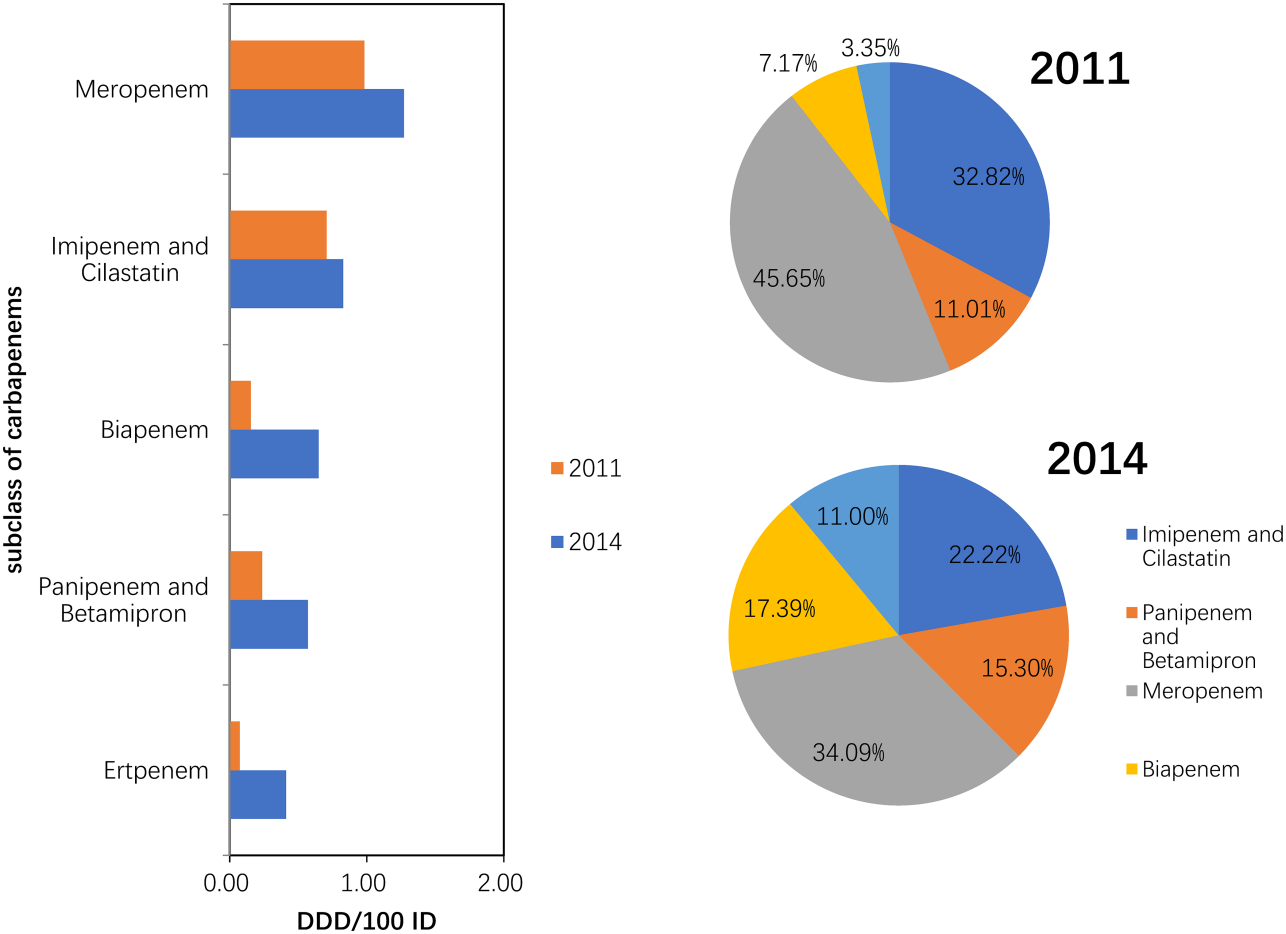
Changes in the patterns of carbapenem use in hospital care between 2011 and 2014 (n = 151 hospitals).

**Fig 6:**
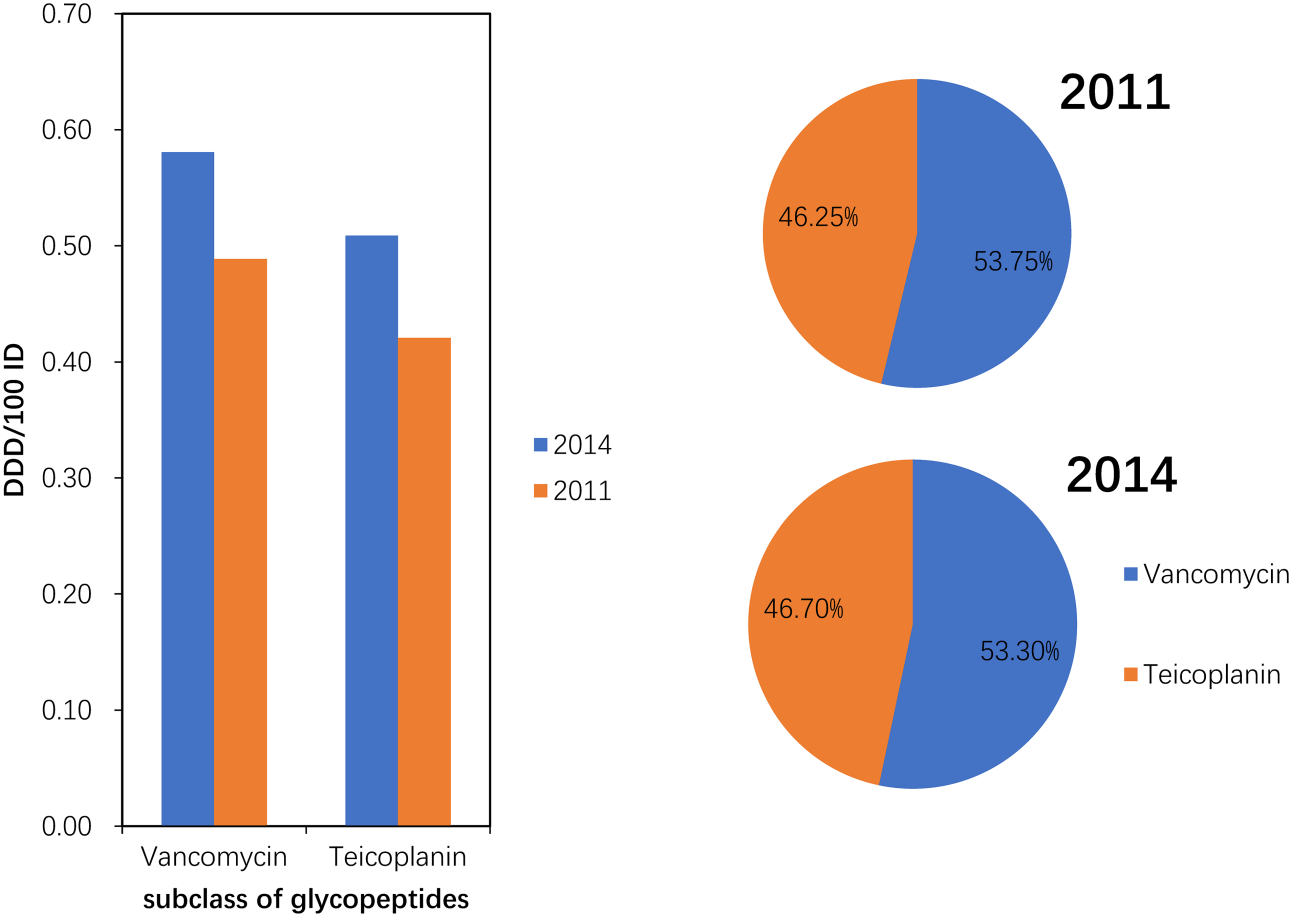
Changes in the patterns of glycopeptide use in hospital care between 2011 and 2014 (n = 151 hospitals).

Compared with 2011, the consumption of carbapenem (J01DH) antibiotics was significantly increased (P = 0.043) in 2014. A large (more than 100%) increase was observed in the use of panipenem/betamipron, biapenem and ertapenem, with the proportions of each drug accounting for more than 10% of the total carbapenem consumption (Fig 5). The consumption of vancomycin and teicoplanin increased in 2014 compared with that in 2011, and the proportion of each drug was relatively stable (Fig 6).

### Proportions of use within the main antibiotic classes in 2014

As shown in Table 2, cephalosporin (including carbapenems and aztreonam) use accounted for more than half of all antibiotic consumption in different regions in 2014, with a range of 58.6% (NWC) to 50.74% (CC). The proportion of penicillin use ranged from 12.51% (NC) to 17.14% (SWC) in all areas. Consumption of quinolone ranged from 16.74% (EC) to 10.59% (NEC). A high (10% or more) proportion of consumption of other antibiotics (J01A/B/E/G) was revealed in NC (12.56%).

**Fig 7:**
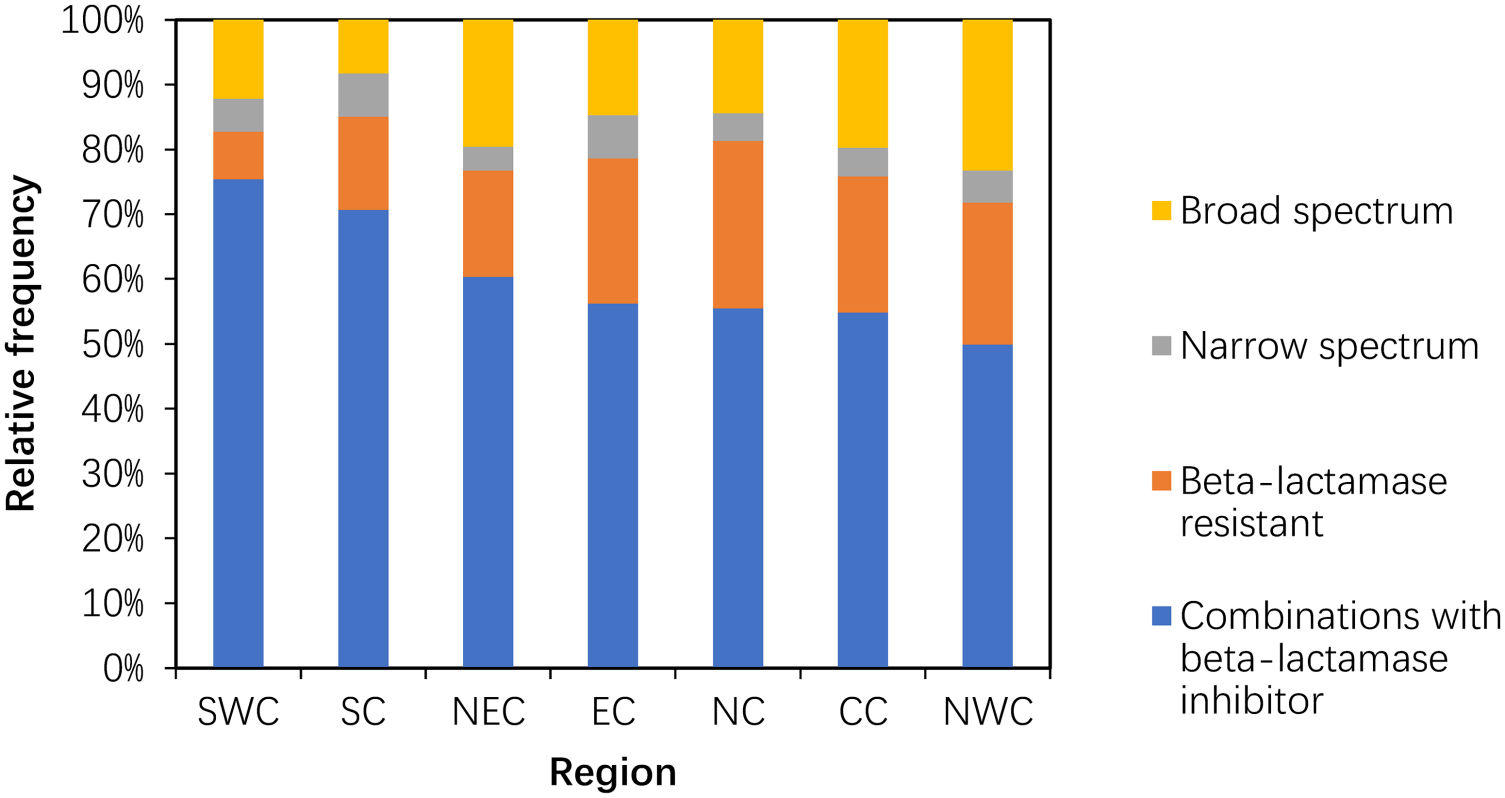
Proportional use within penicillins in hospital care among seven regions in 2014.

Within the penicillins (J01C), the use of combinations of penicillins accounted for most (50% and more) of the penicillin consumption in all areas, and the most frequent use occurred in SWC. The proportion of broad-spectrum penicillins was high (20% and more) in NWC, with a range between 20% and 10% in five areas, and was low (less than 10%) in SC. The proportion of beta-lactam-resistant penicillins was high (20% and more) in four areas (NC, EC, CC and NWC) and ranged from 7% to 17% in the remaining areas (Fig 7).

**Fig 8:**
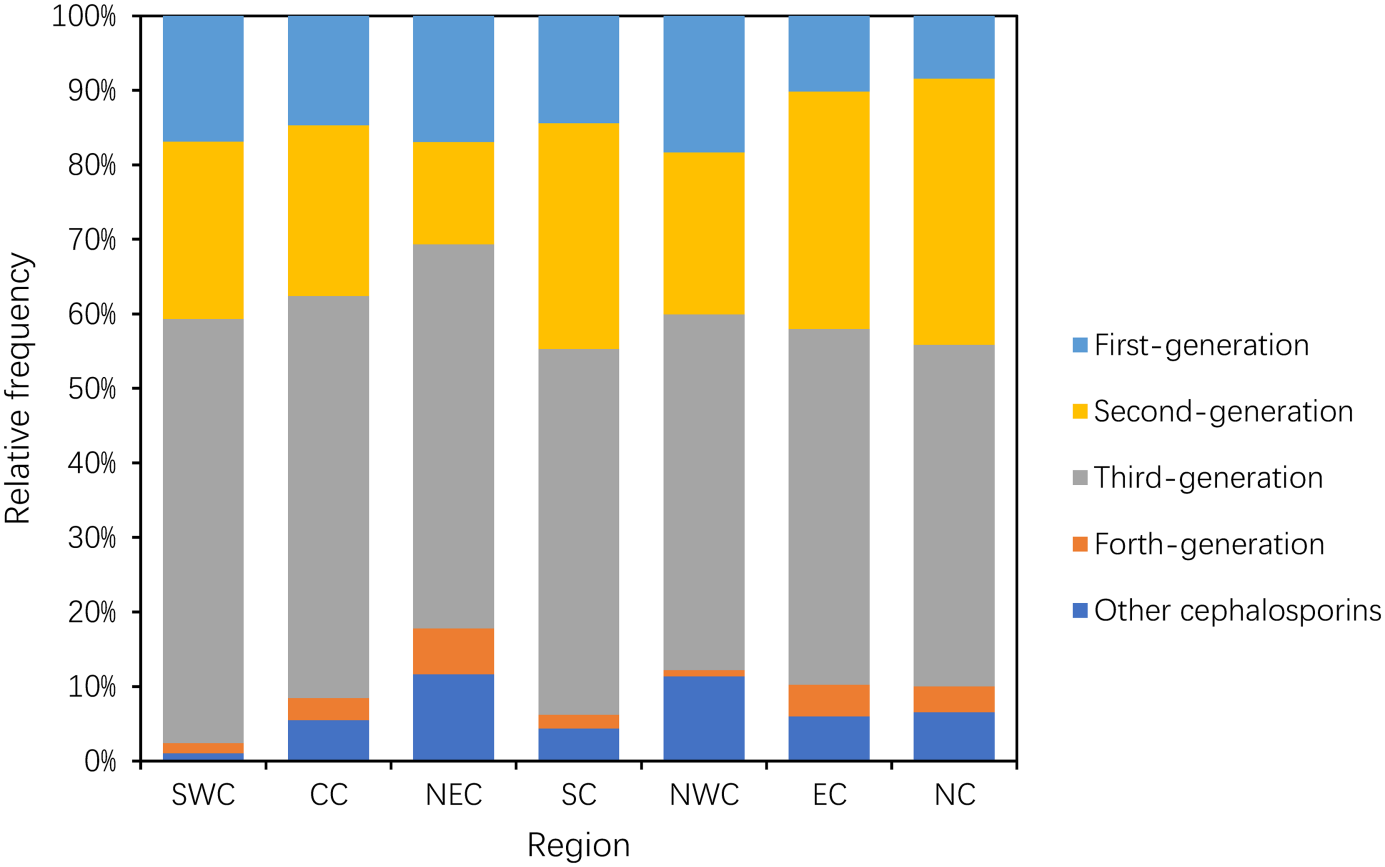
Proportion use within cephalosporins in hospital care among seven regions in 2014.

Within the cephalosporins (including other cephalosporins), the proportion of the third-generation cephalosporins accounted for more than 45%; SWC had the highest proportion (56.91%), and NC had the lowest proportion (45.81%). The proportion of the second-generation cephalosporins was 30% or greater in NC, EC and SC; ranged between 21% and 24% in NWC, SWC and CC; and was 13.37% in NEC. The lowest proportion of the first-generation cephalosporins occurred in NC (8.39%), and the remaining areas had a proportion ranging from 10.12% to 18.23%. The relative frequency of the consumption of the fourth-generation cephalosporins ranged from 0.81% in NWC to 6.19% in NEC. The proportion of carbapenems and aztreonam was greater than 10% in NEC and NWC but was low in SWC (1.09%) (Fig 8).

**Fig 9:**
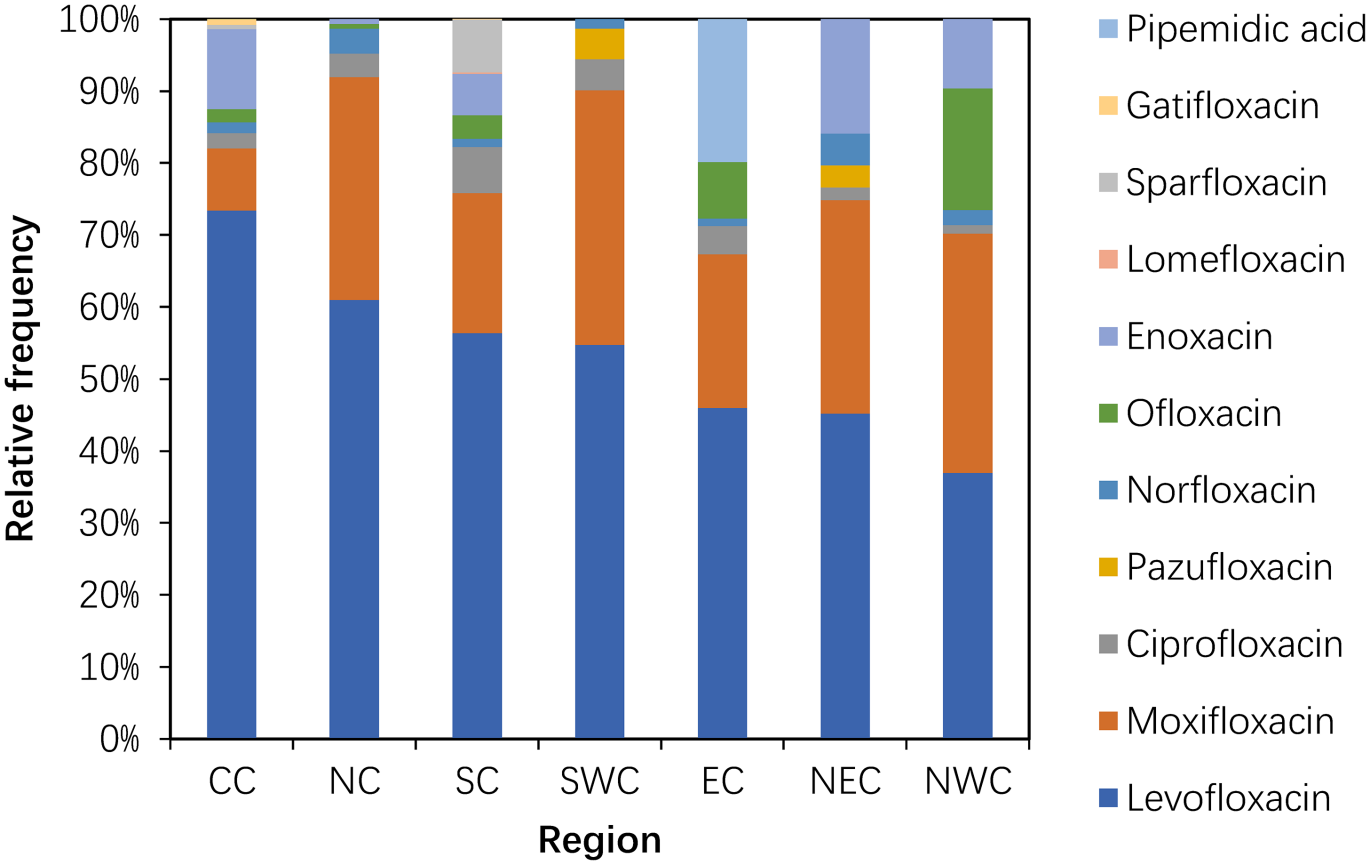
Proportions use within quinolone in hospital care among seven regions in 2014.

Within the quinolones, the proportion of levofloxacin accounted for the majority in each area and was greater than 50% in CC (73.41%), NC (60.99%), SC (56.38%) and SWC (54.74%). The proportion of moxifloxacin was greater than 30% in SWC (35.39%), NWC (33.22%) and NC (30.96%) and less than 10% in CC (8.66%). The primary use of ofloxacin occurred in NWC, and the highest proportion of pipemidic acid use occurred in EC. Enoxacin was mainly used in NEC, CC and NWC (Fig 9).

**Fig 10:**
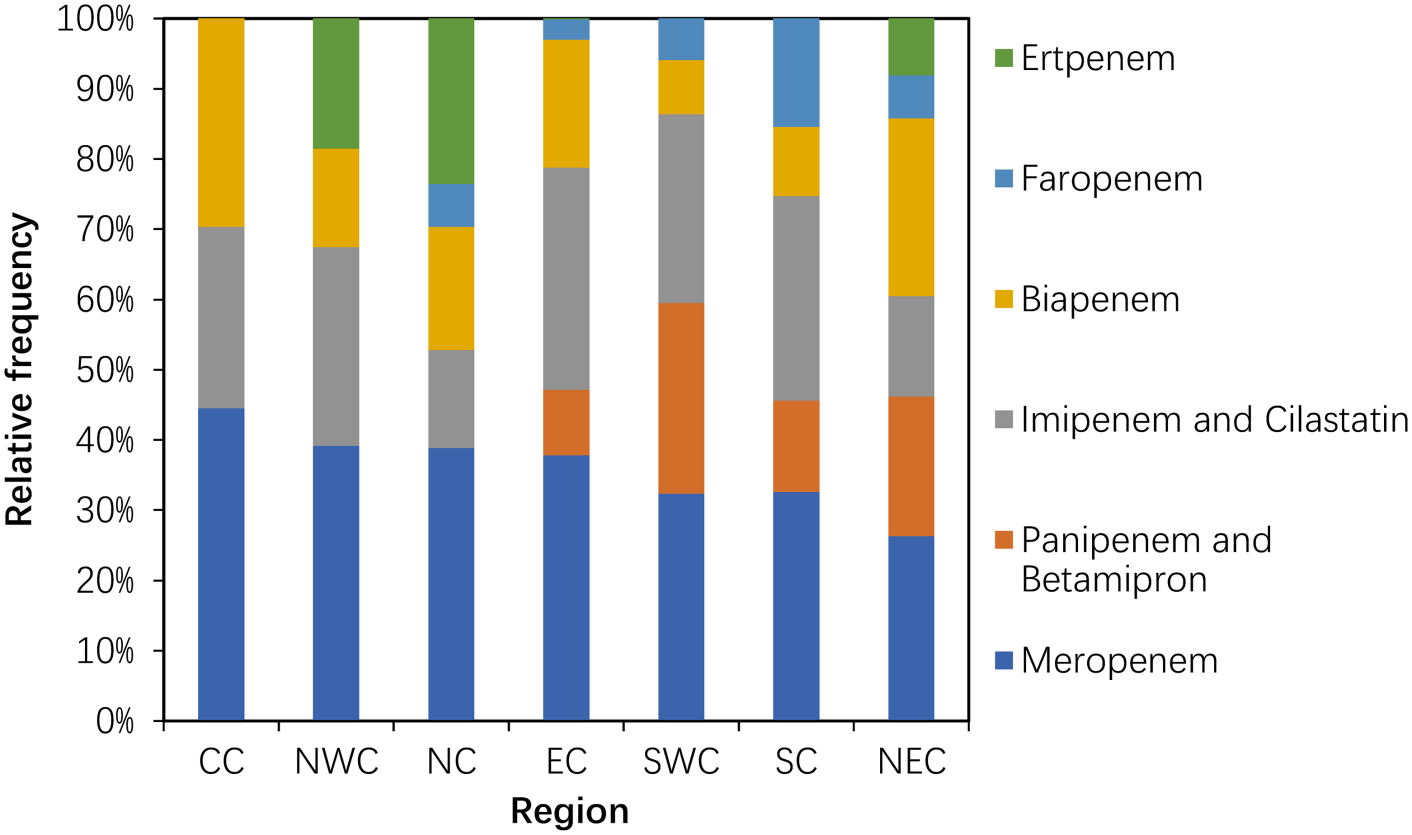
Proportions use within carbapenem in hospital care among seven regions in 2014.

Within the carbapenems used in the participating hospitals, meropenem use accounted for the highest consumption in each area, with a high proportion (40% and more) in CC and a low proportion (less than 30%) in NEC. Panipenem and betamipron were used only in SWC (27.18%), NEC (19.90%), SC (13.025) and EC (9.34%). Ertapenem and faropenem were used only in NC (23.53%), NWC (18.55%) and NEC (8.07%) (Fig 10).

**Fig 11:**
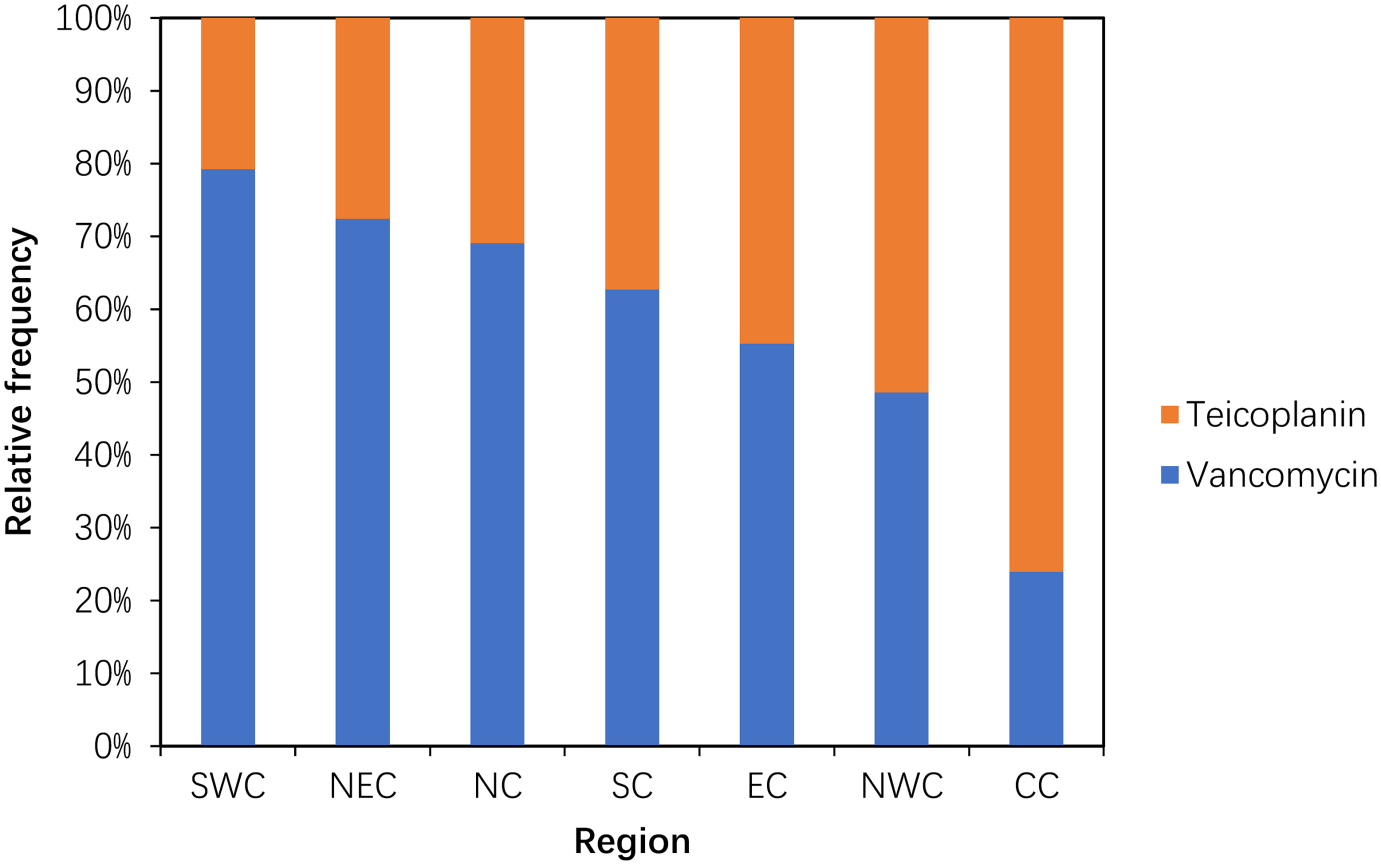
Proportional use within glycopeptide in hospital care among seven regions in 2014.

Within the glycopeptides, the proportion of vancomycin use was high (75% or greater) in SWC and low (less than 25%) in CC. The use of teicoplanin was substantial (75% or greater) only in CC (Fig 11).

### Consumption of antibiotics in hospitals in different regions in 2014

**Fig 12:**
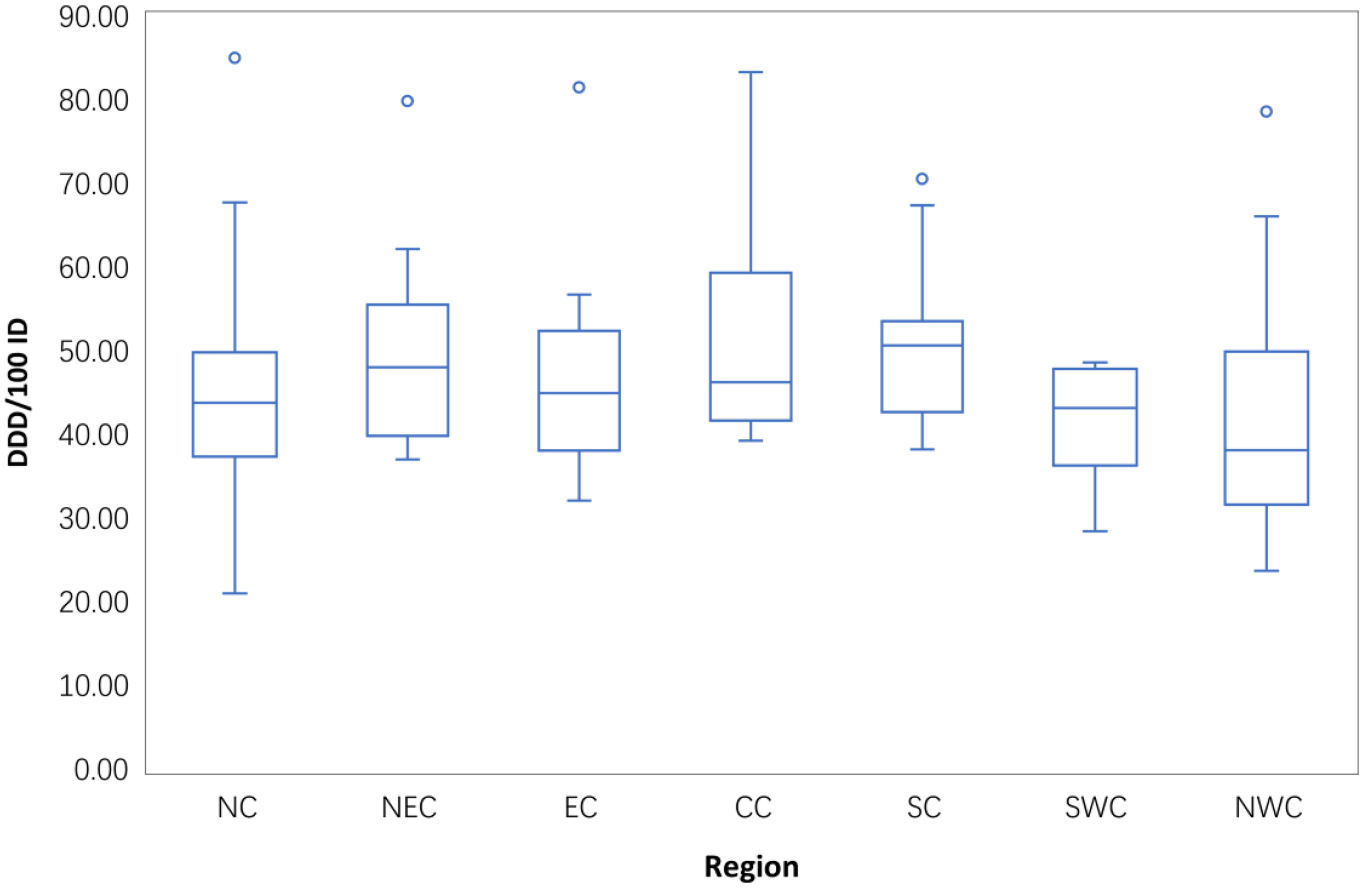
Regional differences in total antibiotic consumption in the 151 hospitals in 2014.

Boxplots of the antibiotic consumption in 151 hospitals in the different areas in 2014 are provided in Fig 12. The total hospital antibiotic use in 2014 differed significantly across different areas (P = 0.014). The median use of antibiotics in each area showed a different distribution; the median antibiotic consumption in NWC was less than 40 DDD/100 ID, whereas the consumption in the remaining regions ranged between 40 DDD/100 ID and 50 DDD/100 ID with the exception of SC (50.31DDD/100 ID). Wide variation was observed in the hospital use of antibiotics in NC (range from 21.68 DDD/100 ID to 85.06 DDD/100 ID) and NWC (range from 23.71 DDD/100 ID to 78.61 DDD/100 ID).

## Discussion

To the best of our knowledge, this study is the first to estimate regional differences and compare the evolution of antibiotic consumption in hospital care across China. We showed that antibiotic consumption by inpatients in public tertiary hospitals decreased substantially (37.93% decrease between quarter 1 of 2011 and quarter 4 of 2012). The trends were similar to previous studies in China[7, 15–17]. These findings reflect the impact of the national 3-year action conducted by the Chinese government after 2011. The Chinese government plays a substantial role in controlling antibiotic usage in public hospitals, which may be related to the public hospital management system that is governed by bureaucratic rules in China[6].

A previous article showed that broad-spectrum antibiotics were preferred in China from 2005–2012[16], our study noted that this preference was still present in 2014. Although the consumption of total antibiotics decreased, the proportion of broad-spectrum antibiotic use remained stable or increased during the study period, especially cephalosporins (J01D, mainly second- and third-generation cephalosporins), which accounted for 50% of antibiotic use in each region of China in 2014. In contrast, penicillins (J01C) are always the most frequently used antibiotics in EU countries[18], the Republic of Korea (ROK)[14]and New Zealand[19]. The reasons for the large proportion of broad-spectrum antibiotic utilization in Chinese tertiary hospitals were likely the following: many Chinese doctors regard broad-spectrum antibiotics as the most efficient treatment to address many infections and relied on empirical treatment[16], commercial promotions and higher resistance rates in China[5]. We could not explain the fast growth of the use of other cephalosporins (mainly cefminox) in this study because we did not have information on the inpatient diseases. Therefore, the real influencing factors need to be further researched.

We found that the broad-spectrum penicillins (e.g., amoxicillin and sulbactam, ampicillin and sulbactam and amoxicillin and clavulanate) were used more frequently than the narrow-spectrum penicillins, which was similar to observations in EU countries[18] and the ROK[14]. Additionally, the increase in exposure to beta-lactamase-resistant penicillins (e.g., flucloxacillin, cloxacillin and nafcillin) may be due to the development of antibiotic resistance[11, 20] and more treatment courses, or to higher does and longer durations per treatment course.

The number of quinolones was reduced for five subclasses (lomefloxacin, rufloxacin, gemifloxacin, fleroxacin and pefloxacin), and levofloxacin (J01M12) remained the frequently used drug among the quinolines in 2014. The probable reason was that quinolones were severely restricted by the MOH due to the increasing rate of bacterial resistance in 2008[21], and the 3-year action required that antibiotic procurement be restricted to 50 agents in tertiary hospitals. Therefore, many hospitals gradually reduced quinolone use. According to data from the Chinese Antimicrobial Resistance Surveillance System (CRASS) in 2015, the prevalence of fluoroquinolone-resistant *E*. *coli* ranged from 43.8%-67.2% in various provinces across China and was higher in the NE than in the SW region[11], which increased the medical burden on both medical institution and patients.

We also found that the consumption of carbapenems (27.03% increase between 2011 and 2014) and glycopeptides (18.48% increase between 2011 and 2014) substantially increased during the study period. Significantly increasing trends were observed for the carbapenems (increased by 64.58%) and glycopeptides (increased by 29.17%) in a nationwide descriptive epidemiological study in the Republic of Korea from 2008–2012[14]. Furthermore, increased consumption of these two drugs has been observed in many countries (e.g., USA, France, Germany, India and the UK) from 2001 to 2010. This could be explained partly by the rise in the global burden of MRSA and ESBL-producing Gram-negative bacteria[3]. These two classes were used only in hospitals in China, and most were used in tertiary hospitals because these two classes of antibiotics required a specialist for their administration and use[22]. A second possible explanation is the increase in the ESBL-producing bacteria identified in the surveillance of bacterial resistance in China[23]. A third possible reason is economic incentives; physicians’ bonuses are frequently tied to drug revenues[6]. The fourth possible reason was that these general tertiary hospitals were mostly local health centres that absorbed a large number of critically ill patients with serious diseases. A study of national antimicrobial resistance surveillance reported that a marked increase in resistance to imipenem and meropenem, from 2.4 to 10.5 and from 2.6 to 13.4%, respectively, was seen in *Klebsiella pneumoniae* from 2005–2014[23]. The relationship between hospital carbapenem use and resistance in *Klebsiella pneumoniae* needs to be analysed further.

The aggregated data from the CAS during the study period revealed an antibiotic consumption pattern in different areas across China that was similar to the ATC-3 classification. Considering the different socio-economic conditions and bacterial resistance, the MOH required health authorities of different areas to develop antibacterial drug lists that met local conditions[8]. In addition, differences in the use intensities and patterns of antibiotics across regions may be due to the natural environment, socio-economic determinants and bacterial resistance. This point should be made clear while exploring the relationships between specific resistance patterns and consumption of particular classes of antibiotics within each region.

One of the targets of the 2011 intervention in hospital care was that total antibiotic use should be limited to less than 40 DDD/100 ID[9]. We showed differences in antibiotic consumption in hospital care across seven regions in 2014 in this study. These differences might be related to differences in hospital size, the number of hospital beds per 100,000 inhabitants[24], the average length of stay[25], demographic characteristics of inpatients in different areas and the distribution of medical resources, especially the lack of skilled doctors in rural areas, which are mainly served by public tertiary hospitals[16]. MacKenzie *et al*. [26]found a significant relationship between the number of antibiotics used and total antibiotic use in 139 hospitals from 30 countries in Europe. This relationship in Chinese hospitals needs to be study further. We found that some hospitals failed to meet the policy goals, possibly because the implementation of antimicrobial stewardship programmes in hospitals was different.

CAS was the first surveillance project by the MOH to monitor antibiotic consumption online at the national level in China. The monitored data provided credible and systematic information regarding antibiotic consumption in hospitals and enabled health authorities at national and regional centres to make scientific decisions[27]. The results from the present study reflect the recent status of antibiotic consumption in hospitals in China and can be used to implement antimicrobial stewardship programmes to prevent the development of antimicrobial resistance in different regions.

There are several limitations to the study. First, valid data concerning antibiotic use were collected from 2011–14 due to the restriction of data availability. Because we could not obtain data prior to 2011, we could not compare the data before and after the 3-year national action. Second, the original data were collected only from public tertiary hospitals because many information systems of secondary hospitals did not have access to the CAS, and the data were not updated in a timely manner. Third, some agencies were not covered by the surveillance system, such as village clinics and township health centres. Fourth, the CAS collected data only for inpatients; therefore, we could not analyse the relationships between antibiotic consumption in ambulatory and hospital care. According to a study in Europe, the basic characteristics of use (extent and nature) are strongly correlated between the two healthcare settings within each country[28]. Fifth, many factors contribute to different patterns of antibiotic consumption in different regions (e.g., the severity of the patient’s disease), but the data collected in this study are limited, and thus the reasons merit further attention. Irrational antibiotic use has been reported to be common in rural areas in China[29], and purchasing antibiotics without a prescription from community pharmacies and retailers remains common in China but is not monitored[30]. Furthermore, the data used in this study do not indicate whether the changes in antibiotic consumption resulted in less inappropriate antibiotic use.

According to the report of Chinese Antimicrobial Resistance Surveillance System (CARSS) from 2011–2015, the prevalence of MRSA, third-generation cephalosporin-resistant *Escherichia*. *coli* and third-generation cephalosporin-resistant *Klebsiella pneumoniae* dropped from 51.8%, 71.8%, and 62.3% to 35.8%, 59.0% and 36.5%, respectively. The prevalence of carbapenem-resistant *Pseudomonas aeruginosa* has also declined, and the resistance of *Enterococcus faecium* to vancomycin remained relatively stable[11]. In the future, to control the misuse and overuse of antibiotics in hospitals, Chinese hospitals should undertake antibiotic stewardship programmes, which include antibiotic committees (multidisciplinary and with at least an infectious disease physician, a microbiologist, a hospital pharmacist and clinicians from the major disciplines), antibiotic guidelines, guideline implementation and quality indicators[31]. To continue and expand studies of antibiotic consumption in hospital care, cooperation between public health officials, healthcare providers, professional associations of microbiologists, drug utilization researchers and hospital pharmacists will be necessary.

## Conclusion

We revealed a significant reduction in antibiotic use in public tertiary hospitals in China after the national initiative that aimed to reduce the misuse and overuse of antibiotics. Although the volume and intensity of total antibiotic use decreased, the patterns of antibiotic use in 2014 were not optimal. We also found that the consumption patterns of antibiotics for systematic use (J01) in different regions across China were similar and that the most used antibiotics were the cephalosporins (J01D). The subclasses of each antibiotic group were used differently in the seven regions, and the intensities of the antibiotic use in hospitals in different regions were significantly different. These findings provide useful information for the improvement of the rational use of antibiotics. However, we did not analyse the specific reasons for these differences because we did not collect determinants of drug use (including information on users, prescribers and drugs and the trends of antimicrobial resistance in hospitals). Thus, further studies about the factors that determine prescribing behaviour in hospitals are needed.

## Supporting information

S1 Table Total antibiotic consumption in 151 hospitals in China (2011–2014). (DOCX)

S2 Table Consumption of the main classes of antibiotics in 2011 and 2014. (XLSX)

S3 Table Use of the main classes of antibiotics in different regions in 2014. (XLSX)

S4 Table Consumption of antibiotics in hospitals in different regions in 2014. (XLSX)
