# Supplementary material for: Consumption of antibiotics in Chinese public general tertiary hospitals (2011-2014): Trends, pattern changes and regional differences
Source: PLoS One. 2018 May 3;13(5):e0196668. doi: 10.1371/journal.pone.0196668 (PMC5933762; doi:10.1371/journal.pone.0196668)
Supplement: S1 Table — (DOCX) [file pone.0196668.s001.docx]

| season | Antibiotic use (DDD/100ID) |
| --- | --- |
| Q1/2011 | 75.86 |
| Q2/2011 | 65.93 |
| Q3/2011 | 59.27 |
| Q4/2011 | 56.65 |
| Q1/2012 | 53.44 |
| Q2/2012 | 49.75 |
| Q3/2012 | 48.96 |
| Q4/2012 | 46.32 |
| Q1/2013 | 51.18 |
| Q2/2013 | 46.33 |
| Q3/2013 | 45.94 |
| Q4/2013 | 47.06 |
| Q1/2014 | 49.21 |
| Q2/2014 | 45.49 |
| Q3/2014 | 46.60 |
| Q4/2014 | 47.09 |

S1 Table. Total antibiotic consumption in 151 hospitals in China (2011-2014)
